# Prediction of β-barrel membrane proteins by searching for restricted domains

**DOI:** 10.1186/1471-2105-6-254

**Published:** 2005-10-14

**Authors:** Oliver Mirus, Enrico Schleiff

**Affiliations:** 1 Botanisches Institut der Ludwig-Maximilians-Universität München, Menzinger Str. 67, 80638 München, Germany

## Abstract

**Background:**

The identification of β-barrel membrane proteins out of a genomic/proteomic background is one of the rapidly developing fields in bioinformatics. Our main goal is the prediction of such proteins in genome/proteome wide analyses.

**Results:**

For the prediction of β-barrel membrane proteins within prokaryotic proteomes a set of parameters was developed. We have focused on a procedure with a low false positive rate beside a procedure with lowest false prediction rate to obtain a high certainty for the predicted sequences. We demonstrate that the discrimination between β-barrel membrane proteins and other proteins is improved by analyzing a length limited region. The developed set of parameters is applied to the proteome of *E. coli *and the results are compared to four other described procedures.

**Conclusion:**

Analyzing the β-barrel membrane proteins revealed the presence of a defined membrane inserted β-barrel region. This information can now be used to refine other prediction programs as well. So far, all tested programs fail to predict outer membrane proteins in the proteome of the prokaryote *E. coli *with high reliability. However, the reliability of the prediction is improved significantly by a combinatory approach of several programs. The consequences and usability of the developed scores are discussed.

## Background

Genomes of numerous organisms are sequenced. Computer-assisted assignment of coding regions of the organism of interest is the first important step for the understanding of the complex proteomic network [1]. Even though the quality of such predictions will be satisfying in future, the knowledge of the sequences of the gene products alone will not provide insight into their function or localization in the cell. In addition, the emphasis has switched from the study of individual molecules to a large-scale, high-throughput examination of genes and gene products of an organism with the aim of assigning their functions [2] and placing them into the complex biochemical networks. One kind of information comes from the structural classification of gene products. Since genome and proteome projects result in a rapid increase of information, the biochemical analysis has to be accomplished by *in silico *predictions [3]. One of the central questions is the localization of proteins since up to 50% of the proteins of a cell have to traverse at least one membrane in order to reach their place of function within organellar compartments [4]. In the past, several prediction programs have been developed for this purpose [5]. However, the analysis of the intracellular localization of a protein is not only limited to the question to which organelle the protein is targeted. One important functional aspect is the distribution of the protein within this cell compartment. For some sub-organellar compartments like thylakoids predictions can be performed based on the targeting signal [6].

However, to date no differentiation of the signal is found for most sub-organellar localizations. So far, various approaches exist to identify helical transmembrane proteins [7,8]. More recently, however, the focus was shifted slightly to include the prediction of β-barrel membrane proteins. Initially, structure prediction was applied with reasonable success when proteins already known to form β-barrel structures were modeled [9]. Now, four alternative directions are used in order to newly identify β-barrel proteins out of a genomic/proteomic data set. In the first approach, sequence profile based HMMs for predicting β-barrel membrane proteins were developed [10-12]. The second methodology is based on the alternating hydrophobicity as a measure for β-barrel transmembrane segments [13]. Thirdly, the structural data of the β-barrel membrane proteins were statistically analyzed and certain criteria developed for a linear prediction [14,15]. The fourth methodology is based on a modified k-nearest neighbor algorithm of the whole sequence amino acid composition [16,17]. Recently, the combination of several independent procedures for β-barrel membrane protein prediction [18,19] or their combination with other procedures, e.g. signal sequence prediction [15,19], was employed to improve the prediction quality.

To evaluate the performance of the developed procedures, test pools are commonly used to derive parameters that discriminate proteins of interest from those of structurally different classes. To avoid an overrepresentation of certain protein families, sequences are removed until each pair of proteins in the pool shares a degree of identity below a certain user defined threshold. Several algorithms have been published to solve this global optimization problem [e.g. [20-22]]. Based on such test pools a comparison of the above mentioned strategies revealed a differential behavior. For example, Deng and co-workers [23] demonstrated that the linear predictor has a very low false positive but a high false negative rate. In contrast, a broader comparison of the predictors performed by Bagos and co-workers [24] manifested that the different predictors perform with a similar quality of about 25% false prediction.

We now improved the linear prediction by implementing new parameters and alterations of the previously established parameters based on test pools to increase the reliability and to avoid manual selection. Here, our main goals were to maintain a very low false positive rate and to reduce the high false negative rate of about 51% as reported by Deng and co-workers [23] for the original prediction method by Wimley [14]. We present parameters for β-barrel membrane protein identification and their prediction performance on the proteome of the prokaryote *E. coli*.

## Results

**Figure 1:**
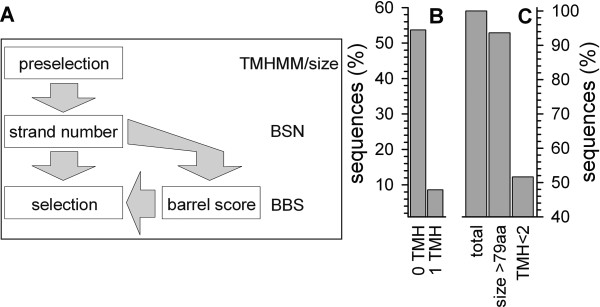
**The selection criteria**. (A) Schematic view of the scores for prediction. (B) The amount of sequences in all test pools with 0 or 1 transmembrane helix (TMH) predicted by TMHMM. (C) The amount of sequences in the pools with NOM proteins (lane 1) greater than 79 amino acids (lane 2) and with less than 2 predicted transmembrane helices (lane 3).

First, the published set of parameters (Fig. 1A) [14,15] was used to analyze proteomic data. The parameter set is defined as following (Table 1): the statistical values of the probability for an amino acid to be present in either the lipid tail or head group region and facing the membrane or channel interior in membrane-inserted β-strands were taken from Wimley [14]. The β-strand length for the calculation of the exact β-strand score (EBSS, see Methods) and for the hairpin score (HPS) was chosen to be 10 amino acids following the original argumentation [14]. The loop length for calculation of the HPS was set to cover the range from the initial strand up to 14 amino acids distance. Previously, a minimal loop length of four [14] or five amino acids [15] was considered. For the calculation of the β-barrel score (BBS) of a protein the selection criterion for the HPS value was set to >6.0. For the calculation of the β-strand number (BSN) all independent EBSS peaks >2.0 were counted. For the final selection of β-barrel proteins, all proteins with a BBS of at least 0.7 and all proteins with a BSN above 13 were collected [15]. For comparison, the amount of sequences predicted to be β-barrel membrane proteins of the *E. coli *proteome using this parameter set was analyzed. Employing the original proposed set – selecting all proteins with a BBS greater than or equal to 1.0 – about ~4% proteins of the *E. coli *proteome were selected [14]. Applying the modified set [15], about 12.2% of all sequences of the *E. coli *proteome were predicted to form a membrane inserted β-barrel. The larger number observed with the new criteria might represent an increase of the false positive prediction. Especially the introduction of the BSN criterion, even though essential for a reduction of the false negative rate of β-barrel membrane protein selection in eukaryotic background, revealed the prediction of soluble proteins [15]. As a consequence, we have focused on the BBS and BSN to refine the prediction procedure without altering the calculation of the EBSS.

**Table 1:** Summary of the parameters for β-barrel prediction

score^a^	definition of the parameter^b^	value used^c^
EBSS	β-strand length	10 aa
	Core region	4 aa
BSN^d^	Number of individual peaks	EBSS >2.0
	β-strand length	10 aa
HPS	β-strand length	10 aa
	Loop length	0–14 aa^d^
BBS	HPS cut off	>6.0
BBS275^d^	HPS cut off	>6.0
	Sliding window	275 aa
BSHS225^d^	EBSS cut off for strands	>2.0
	Sliding window	225 aa

For detailed descriptions see text. ^a ^... The different intermediate scores necessary for final calculation of the β-barrel score; ^b ^... the scores are calculated by various physicochemical assumptions; ^c ^... the parameters for the different scores were used as suggested by Wimley, if not stated differently [14]; ^d ^... described in here

### Preceding parameters for selection

Three global rules were defined for all subsequent predictions. First, at least 8 transmembrane β-strands [25] are required to form a β-barrel embedded in a lipid bilayer. In general, the average length of a single transmembrane β-strand is 10 amino acids [14]. Therefore, a membrane inserted β-barrel contains at least 80 amino acids and consequently the first cut off defined is a rejection of all sequences below 80 amino acids (Fig. 1C, Table 2). Second, we found that a pre- or post-selection by TMHMM [26] improves the performance. Previously was suggested, that two predicted transmembrane helices are an indication of a helical membrane protein [27]. Therefore, sequences with more than one predicted transmembrane helix are considered as helical anchored and rejected from the β-barrel prediction (Fig. 1B and 1C; Table 2). Here, the reliability of this step is defined by the false positive rate of TMHMM in regard to β-barrel membrane proteins. However, screening the PSort OM (outer membrane) test pool, PilQ, probably a β-barrel membrane protein involved in the assembly or modification of pili [28], is – besides two small cysteine-rich proteins – the only protein with more than one predicted transmembrane helix. Third, in order to select a sequence as a β-barrel membrane protein all scores defined in the following have to be above zero.

**Table 2:** The new parameter set for linear prediction

Parameter	old^a^	new^b^	new^c^	new^d^
aa	-	>79		
TMHMM	<1	<2		
BSN	>13	>10		
BSN/aa	-	>0.026		
BBS275	-	>2.5	>1.35	>1.35
BSHS225	-	>0.12	>0.11	>0.04
BBS	>0.7	-	-	-

Shown are the parameters used in here for linear prediction (new). They are compared to previously used parameters (old). ^a ^...values as previously described [15], ^b ^...values for a prediction with 0% false positive rate (BBS275 OR BSHS225), ^c ^...values for prediction with lowest false prediction rate considering independent selection by BBS275 and BSHS225 (OR), ^d ^...values for prediction with lowest false prediction rate considering dependent selection by BBS275 and BSHS225 (AND).

### Control of the incorporated β-strand number

One of the selection criteria for membrane β-barrel proteins was based on the β-strand number (BSN) of the proteins [15]. The previous BSN was calculated by selecting each individual region with EBSS values above 2.0. Hence, in a stretch of 10 amino acids considered as β-strand several counts can exist if values above 2.0 are separated by values equal or less than 2.0. We changed this algorithm as follows. The first predicted strand now starts at the amino acid with the highest EBSS. The preceding and succeeding nine amino acids are excluded from the search for the highest EBSS in the remaining values to account as starting amino acid of another strand. The β-strand selection procedure stops when no EBSS above 2.0 is left or all amino acids have been assigned to β-strand or pre-β-strand regions.

**Figure 2:**
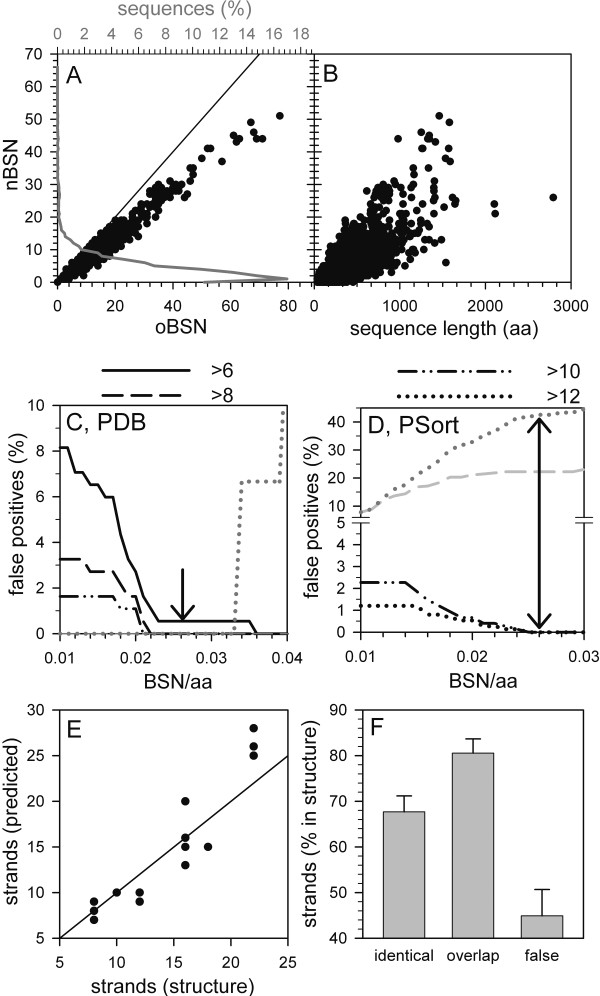
**The BSN selection**. (A) The relationship between BSN calculated by the old and new procedure is shown for the sequences of *E. coli *(circle, bottom x-axis). The percentage of sequences with a certain BSN is shown as line plot (top x-axis). (B) The sequence length (in amino acids) dependence of the new BSN for sequences of *E. coli *is shown. (C-D) Sequences with a BSN value above 6 (solid), 8 (dashed), 10 (dashed-dotted) or 12 (dotted) were selected from the PDB (C) or PSort (D) pools. Subsequently, for the generated sequences pools the percentage of false positive selected sequences from NOM protein pools (black lines) and the false negative selected sequences from OM protein pools (grey lines) in relation to the BSN/aa cut off was determined. (E) The numbers of structurally determined strands and of predicted strands are shown; the line indicates a similar detection value. (F) The amount of strands predicted at identical position (maximum 1 amino acid mismatch; identical), of strands predicted at identical or overlapping position (maximum 5 amino acids mismatch; overlap) and the amount of false negative and false positive predicted strands (false) is shown.

Hence, the number of counted strands is reduced in prokaryotic sequences (Fig. 2A). In addition, analyzing the BSN in regard to the sequence length of the proteins revealed a clear dependence on the amino acid length (Fig. 2B). For most of the sequences of the *E. coli *proteome (Fig. 2A, grey line, about 89%) at least one strand is proposed documenting that a selection by strand appearance alone is not possible. This might be understood as ~2% (2.001*10^11^) of all possible amino acid combinations (1.024*10^13^) analyzing a 10 amino acid window for the EBSS calculation [14] lead to a value above 2.0. Assuming a random distribution within an amino acid sequence, one peak exists about every 60 amino acids. Taking the previous result [15] suggests an even higher number of selected strands leading to the over-representation of large proteins in the first selection, which had to be excluded manually. Analyzing the ratio between BSN and sequence length of the proteins revealed that for most of the sequences of the *E. coli *proteome (56%) one to three membrane inserted β-strands per 200 amino acids are predicted. As the amino acids within sequences are not entirely randomly distributed, the amino acid stretch is found to be a little longer than in the statistical calculation. However, the result is in line with the discussion above and further documents that BSN selection should be controlled by the statistical occurrence of β-strands.

To establish the selection criteria, we analyzed several test pools (described in Methods). First, the percentage of selected sequences from the sequence pools containing non-barrel proteins (Fig. 2C,D, black lines) with different BSN cut offs in relation to the BSN/aa cut off was determined. For these proteins, a BSN selection cut off of 10 in combination with a BSN/aa cut off of 0.026 results in a 0% false positive selection (Fig. 2C,D). This corresponds to one peak of the EBSS above 2.0 every 40 amino acids, which is above the calculated statistical expected frequency (see above). Previously, the existence of at least one transmembrane α-helix per 100 amino acids was defined as cut off for helical transporters [29]. Comparing the length of the β-strand (10 amino acids on average) and of the membrane inserted helix (20–24 amino acids) as well as the number of the inserted membrane segments (statistically membrane β-barrels contain at least twice as many membrane inserted segments compared to helical transporters) supports the defined BSN/aa score of 0.026. This set of parameters leads to a selection of all sequences in the PDB pool of β-barrel transmembrane proteins (Fig. 2C, grey). For the sequences in the PSort pool of OM proteins a false negative rate of at least 43% is achieved (Fig. 2D, grey dotted). However, the further selection by BBS reduces the false negative rate as described below.

We next analyzed whether the new algorithm for BSN calculation could be used for the generation of topological models of the analyzed proteins. As already discussed above, an over prediction of strands is obtained, especially for larger proteins (Fig. 2E). A detailed analysis of the strands predicted (Fig. 2F) revealed a 68% identical positioning allowing one amino acid mismatch and 80% overlapping positioning of the strands requiring at least five amino acids overlap between structural determined and predicted strands. However, the rate of false positioned strands (false negative and false positive selected strands) is 45%. This analysis suggests that the positioning is not as much the problem as the over prediction and the prediction should be combined with the analysis of other physicochemical parameters.

### Development of a new criterion based on the localization of the pore-forming domain

**Figure 3:**
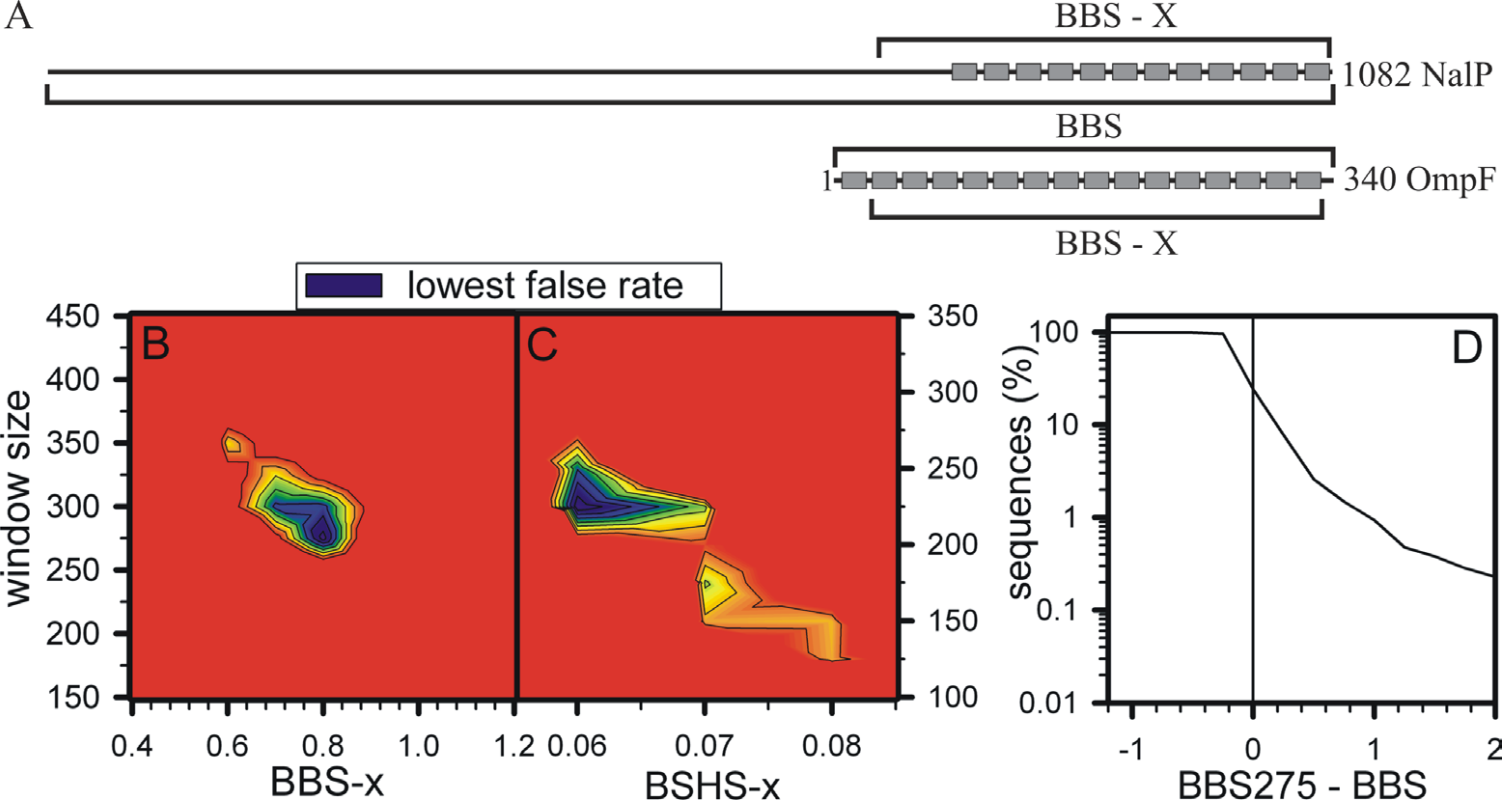
**Analysis of the BBS and BSHS**. (A) The β-strand locations of a *N. meningitidis *(NalP) and an *E. coli *(OmpF) OM protein are shown. The window for calculating the BBS or a domain based BBS (BBS-x) is indicated. (B-C) The false prediction rate for BBS-x (B) or BSHS-x (C) calculation using different amino acid windows and different cut off scores is shown. The regions with the lowest false prediction rates (black) for the three times weighted pool of the NOM proteins is shown. (D) The percentage of sequences above a certain threshold value of BBS275 minus BBS is shown for the sequences of *E. coli*.

Detailed analysis and topological modeling revealed that the pore-forming regions are mostly located within a compact domain (Fig. 3A). Prokaryotic pore-forming proteins/domains are typically of a size between 30 and 35 kDa. The topological models based on the solved structures of OmpF and Nalp are shown as examples (Fig. 3A). Guided by this observation, the scores were now calculated for a defined region of the sequences. We used different scanning windows starting with 75 amino acids as it is below the smallest possible pore unit as discussed above and incremented the window size in steps of 25 amino acids. This window is subsequently moved across the protein and the highest calculated score was selected. For sequences with less amino acids than the window size, the BBS value of the entire sequence is considered. The false positive rates for the combined pools of the non-β-barrel proteins and the false negative rates for the combined pools of β-barrel proteins for each BBS calculation window for different BBS-x (x reflects window size) cut off values were calculated.

*In vivo*, a ratio between soluble and helical membrane proteins of at least 10:1 is expected [27]. It is further reasonable to assume that cells do not contain more β-barrel membrane proteins compared to helical membrane proteins. Indeed, other publications discuss a β-barrel membrane protein content within the entire proteome of 2 to 4% [12,14]. However, our pools represent a ratio of 3.8:1 of NOM (non-outer membrane) to OM proteins. To match the proteomic situation, the false positive prediction rate of the NOM proteins was weighted three times higher than the false negative prediction rate of the OM proteins (Fig. 3B). Hence, a region with lowest false prediction in windows of 250 to 375 amino acids and a BBS-x score of 0.6 to 1.0 could be obtained (Fig. 3B). The lowest false prediction rate was achieved utilizing a window of 275 amino acids with a cut off value of 0.8. Therefore, for the subsequent analysis the BBS is replaced by the BBS in a 275 amino acid window (BBS275). Analysis of the score performance when applied to sequences from *E. coli *(Fig. 3D) shows, that about 70% of all sequences have a smaller BBS275 compared to the old BBS even though the highest value obtained for BBS275 of *E. coli *sequences is similar to the highest BBS value (not shown).

Guided by the development and performance of the new BSN score, we developed and analyzed a new score taking into account the alternating hydrophobicity for each predicted strand. Here, for each predicted transmembrane β-strand its alternating hydrophobicity according to equation 1 (E1) was calculated and multiplied with its EBSS value (E2). The final score BSHS (β-strand based hydrophobicity score) is calculated according to equation 3 (E3). The analysis of the performance of the score was performed as described for BBS275. Here, we identified a window of 225 amino acids as best performing (Fig. 3C). This is in line with a homo-oligomeric complex formation of most of the β-barrel membrane proteins, since strands on the protein-protein interface do not necessarily reveal an alternating hydrophobicity as the strands involved in complex formation are not exposed to the membrane lipids [30].

### Development of scores for the linear predictor

After development of three scores for the linear predictor (BSN, BSHS225 and BBS275) we went on to establish selection procedures. They include the three discussed preceding steps by size, TMHMM [26] prediction and score analysis as discussed above.

**Figure 4:**
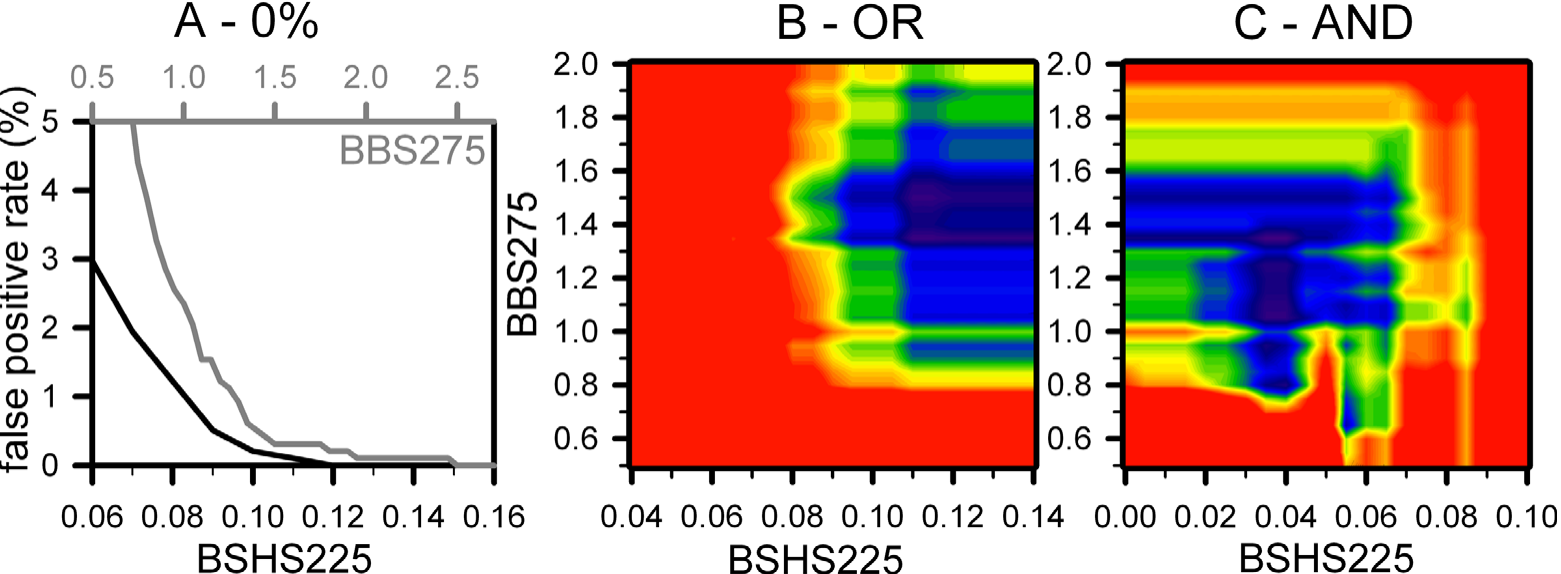
**Score definition for the linear predictor**. (A) The false positive rate for the NOM protein pool in dependence on the BBS275 cut off (grey) or BSHS225 cut off (black) was calculated. (B, C) The false positive selection rate for the NOM protein pool and the false negative rate of the OM protein pool was calculated for a sliding window for BBS275 and BSHS225 considering BSN>10 and BSN/aa>0.026 as preselection rule. The false prediction rate was calculated using a three times higher weight of the false positive rate of NOM proteins. In (B) the false prediction rates of the individual selection by BBS275 and BSHS225 is shown. In (C) the false prediction rate of the dependent selection by BBS275 and BSHS225 is shown.

First, scores for the selection with a low false positive rate had to be established. Hence, the cut offs of BSN and BSN/aa, 10 and 0.026, warrant a low false positive selection according to the analysis of the test pools (Fig. 2C,D; Table 2). Analyzing the BBS275 and BSHS225 distribution of the NOM proteins in the test pools revealed cut off values of 2.5 and 0.12 (independent selection, BBS275 OR BSHS225, Fig. 4A, Table 2), respectively. This procedure selects 62.5% of the OM proteins of the test pools and therefore, the false negative prediction rate is 37.5%.

However, a 0% false positive predictor does not perform with a low false negative prediction rate. We therefore went on to derive scores for the lowest false prediction rate as well. Since the BSN algorithm leads to an over-prediction of strands, we considered the BSN:BSN/aa selection as an initial step and did not alter the cut off values for selection. Subsequently, the selection by the two scores was performed individually by each score (OR selection) or in combination of both scores (AND selection). Again, we weighted the false positive rate of the NOM pools three times higher as the false negative rate of the OM proteins for the discussed reason. Analyzing the selection performance by the individual BBS275 and BSHS225 (Fig. 4B, Table 2) revealed a score cut off combination of 1.35 and 0.11, respectively. BBS275 and BSHS225 in combination (Fig. 4C, Table 2) result in cut off values of 1.35 and 0.04, respectively. For both procedures a false negative rate of 27.5% and a false positive rate of about 1.2% were obtained based on the analyzed test pools.

### Comparison of predictors applied to proteome wide prediction

**Figure 5:**
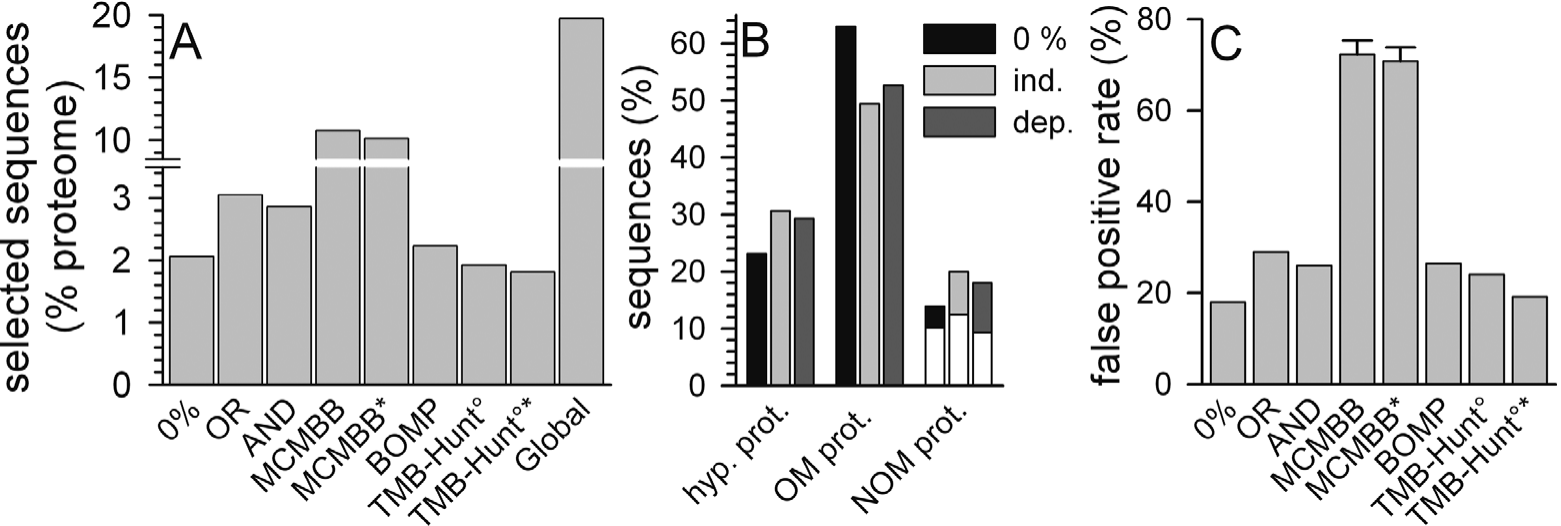
**Identification of β-barrel protein sequences from the *E. coli *proteome**. (A) Sequences were selected from the *E. coli *proteome by the three parameter sets developed (Table 2). The percentage of selected sequences in comparison to the proteome size is shown (bars 1–3). Also shown are the percentage of sequences selected by MCMBB (bar 4), MCMBB filtered by TMHMM (bar 5, MCMBB*), by BOMP (bar 6; please note, that only two sequences were selected by BOMP with αTM >1 according to TMHMM), by TMB-Hunt, BBTM protein score >0 and E-value <1 (TMB-Hunt°, bar 7), by TMB-Hunt, BBTM protein score >0 and E-value <1 controlled by TMHMM (TMB-HUNT°*, bar 8) and by the global procedure (bar 9). (B) The sequences selected by the three procedures proposed in here were analyzed for known or assigned function or localization. The percentage of the sequences either classified as hypothetical, outer membrane, extra-cellular or soluble intracellular is shown. (C) The false positive rate for the three in here generated sequence pools (bars 1–3), for the sequence pool generated by MCMBB (bar 4), by MCMBB controlled by TMHMM (bar 5), by BOMP (bar 6), by TMB-Hunt, BBTM protein score >0 and E-value <1 (TMB-Hunt°, bar 7) or by TMB-Hunt, BBTM protein score >0 and E-value <1, controlled by TMHMM (TMB-Hunt°*, bar 8) is shown.

To further confirm the quality of the developed cut off values we analyzed their performance by prediction of β-barrel proteins from the prokaryotic *E. coli *proteome. Here, 108, 160 or 150 sequences were selected by the cut off values defined for 0%, OR or AND approach. This accounts for 2.1%, 3.1% or 2.9% of the entire proteome, respectively (Fig. 5A). For 83/111/106 sequences (0%, OR, AND) a (proposed) function could be assigned (Fig. 5B). Hence, we found 15/32/27 (0%, OR, AND) sequences not encoding for OM proteins (Fig. 5B). Interestingly, most of the selected NOM proteins are secreted proteins or proteins of the periplasmic space (Fig. 5B, white section). Assuming a similar distribution of the localization of the hypothetical proteins as found for the annotated sequences, we obtain a false positive rate of 18% for the 0% selection, of 29% for the OR procedure and of 26% for the AND procedure (Fig. 5C).

To achieve an impression of the performance quality, we compared our selection with the performance of MCMBB [31], BOMP [19], TMB-Hunt [16,17] and a predictor just based on the global amino acid distribution of β-barrel proteins [32]. MCMBB selects 10% of the *E. coli *proteome (Fig. 5A, MCMBB, 565 sequences). Application of the pre- or post-selection by TMHMM (see above) revealed only a slight reduction of the selected pool (Fig. 5A, MCMBB*, 530 sequences). For both selections we found a very high false positive rate of about 70% (Fig. 5C). Interestingly, for the predictor based on the global amino acid composition an even higher number of sequences was selected (Fig. 5A, Global), which was not drastically altered when post-screened with TMHMM (not shown). Even though it was estimated that about 30% of all proteins are helical membrane proteins [27], it is not considered to be likely that more than 10% of all proteins are β-barrel membrane proteins as discussed above. Therefore, these results raise the question, how reliable scores based on prediction performance on test pools are when transferred to proteome wide prediction.

Using BOMP (Fig. 5A) or TMB-Hunt controlled by the E-value (Fig. 5A, TMB-Hunt°) results in a similar pool size compared to the 0% selection established in here. At default settings BOMP selected 2.23% of the *E. coli *proteome (Fig. 5A, BOMP) with a false positive rate of 26.4% (Fig. 5C, BOMP) and only two proteins with more than one transmembrane helix according to TMHMM prediction (not shown). TMB-Hunt predicted 1.9% of the *E. coli *proteome as integral outer membrane proteins (Fig. 5A, TMB-Hunt°) with a false positive rate of 24.1% when requiring both a BBTM protein score >0 and an E-value <1 (Fig. 5C, TMB-Hunt°). A post-selection by TMHMM reduced the ratio of predicted sequences to 1.8% (Fig. 5A, TMB-Hunt°*) and the false positive rate to 19.2% (Fig. 5C, TMB-Hunt°*).

**Figure 6:**
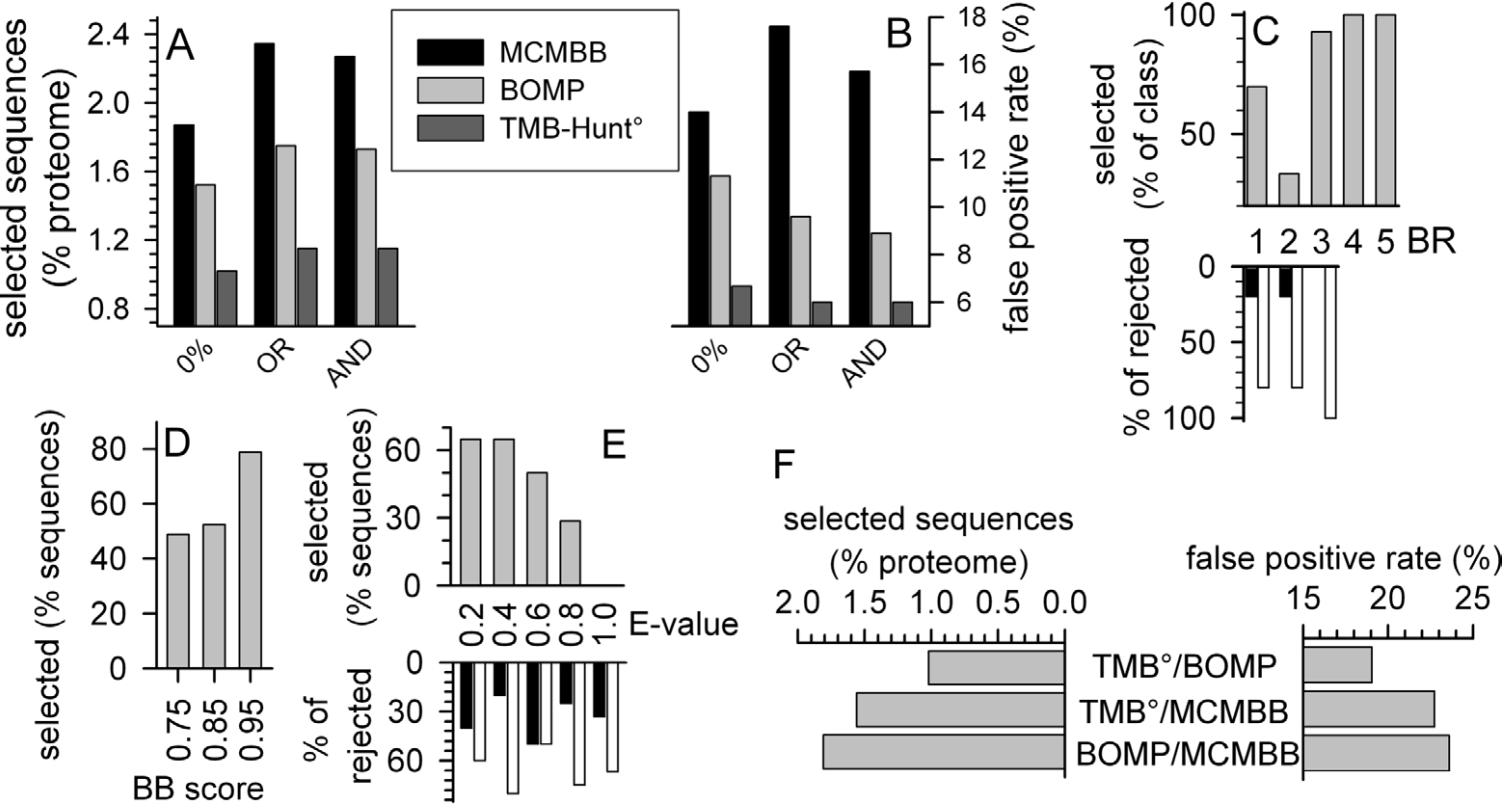
**The performance of the combinatory approach**. (A) The percentage of sequences selected from the *E. coli *proteome by our three methods in combination with MCMBB (black bar), BOMP (light grey bar) and TMB-Hunt, BBTM protein score >0 and E-value <1 (dark grey bar) is given. (B) The false positive rate for combinatory approach performed as under (A) is shown. (C) The percentage of sequences selected by BOMP and our AND selection were analyzed in comparison to the BOMP selection sorted according to the BOMP rank (BR) assigned (top panel, grey). The percentage of the (putative) outer membrane β-barrel proteins (black bar) and (putative) non-outer membrane β-barrel proteins (white bar) in relation to the total amount of rejected sequences is given on the bottom. (D, E) The percentage of sequences selected by TMB-Hunt° and our AND selection were analyzed in comparison to the TMB-Hunt° selection sorted according to the BB score (D) or E-value (E) assigned ({explained in [17]} grey). In (E), the percentage of the (putative) outer membrane β-barrel proteins (black bar) and (putative) non-outer membrane β-barrel proteins (white bar) in relation to the total amount of rejected sequences sorted according to the E-value is given on the bottom. (F) The percentage of the *E. coli *proteome selected by the combinatory approach between TMB-Hunt° & BOMP, TMB-Hunt° & MCMBB, and BOMP & MCMBB is given on the left side. The right side shows the false positive rate as explained in Fig. 5C.

Previously, the combination of several predictors was suggested to improve the selection reliability [18,19]. This strategy allows an increase of the prediction quality as tested on proteomic data of the OM proteome of *Nostoc *sp. PCC7120 [18]. We subsequently analyzed the overlap of our procedures with the output of the other programs. The amount of sequences selected in the overlap of our selection and that of the other programs depends on the size of the selected sequence pools by the individual programs (Fig. 6A). Therefore, this combinatory approach revealed the most sequences in combination with MCMBB and the least number of sequences in combination with TMB-Hunt after E-value selection (Fig. 6A). To see, whether an improvement of the selection quality was achieved, we have analyzed the false positive rate after the combinatory approach. The false positive rate is dependent on the number of sequences in the selected pool (Fig. 6B). Using BOMP, 91 sequences (OR) and 90 sequences (AND) were selected in combination with our procedure showing a false positive rate of 9.6 and 8.6%, respectively (Fig 6A and 6B, light grey bar). The lowest false positive rate of about 6% out of 60 selected sequences was achieved combining our AND or OR method with TMB-Hunt (Fig. 6B, dark grey bar). We next analyzed, whether the same result would be obtained increasing the threshold of BOMP or TMB-Hunt. Analyzing the overlap of the BOMP and the AND selection, we discovered that only sequences of the rank 1–3 were omitted (Fig. 6C). The integral β-barrel score [19] of these proteins is rather low (rank 1 and 3) or even below threshold (rank 2, proteins are only selected by pattern match [19]).

However, simply rejecting all sequences of rank 1–3 from the BOMP selection would not reveal the same result as the overlap procedure. Furthermore, analyzing the rejected sequences we found that most of the sequences rejected from the BOMP prediction are indeed non-β-barrel outer membrane proteins (Fig. 6C, bottom, white). Analyzing the overlap between TMB-Hunt and our AND prediction shows that the BB score [17] does not show any clear preference for rejection (Fig. 6D), whereas all sequences with an E-value above 0.8 were rejected (Fig. 6E). Interestingly, sequences with very low E-values were rejected as well (Fig. 6E). Analysis of the rejected sequences shows that again mostly non-β-barrel proteins are rejected although the amount of β-barrel proteins removed from the selected pool seems to be increased. Finally, we went on to compare the combinatory approach including our predictor with the combinatory approach among the other programs. Again, utilizing MCMBB resulted in a larger number of selected sequences than the combination of TMB-Hunt and BOMP (Fig. 6F, left). However, to our surprise, the false positive rate was not significantly changed in comparison to the individual programs (compare Fig. 6F right and Fig. 5C). This might be explained as all other programs analyze the entire sequence as such, whereas our prediction is based on a defined region of the sequence.

Summarizing, the combination of our procedure with other predictors increased the quality of the performance. However, this improvement is only achieved by a consensus approach of a domain and a full length sequence based predictor.

## Conclusion

The aim of the presented work was to develop better tools or rules for the prediction of β-barrel membrane proteins. In a recent proposal [15] we obtained a significant false prediction of soluble proteins. First, we went on to optimize the developed scores by implementing a new definition of the BSN and a control parameter for this score (BSN/aa, Fig. 2 and Table 2). Further, we analyzed the domain size optimum for β-barrel discrimination (Fig. 3). Here we learned that the best performance was achieved in a window below 300 amino acids. The latter result is in line with the observation that most porins are about 30–35 kDa [25]. Furthermore, for β-barrel proteins of larger size, clustered pore regions were found. For example the structural modeling of FhaC [33], ShlB [34] or Toc75 [15,35] suggests a soluble domain or long loops in the N-terminal region, whereas only the C-terminal portion seems to be involved in pore formation. It might therefore be suggested that an evolutionary prolongation of the membrane β-barrel proteins occurred facilitating the interaction with other proteins or substrates as seen for Toc75 [36]. This result is interesting for the understanding of the evolutionary development of such proteins. It might point to the fact that a minimal structural unit was the starting seed for the development of larger pores as discussed for helical transporters ("hairpin theory") [37]. Finally, we used a combination of an amino acid distribution based score and the theory that membrane facing strands should reveal an alternating hydrophobicity and calculated a combined score in a 225 amino acid window (BSHS). The window size might reflect that strands involved in homo-oligomerisation do not contain as many hydrophobic amino acids compared to those facing the exterior.

By visual inspection of the structures we determined the average sizes of the continuous region exposed to the lipid membrane and of the region containing the β-barrel. The obtained sizes are ~275 and ~325 amino acids on average, respectively. This corresponds quite well to the window sizes determined for BSHS225 and BBS275. Theoretically, the smallest possible β-barrel membrane domain, an 8-stranded β-barrel of about 80 aa length, should represent the optimal screening window size. But as we are not analyzing each protein separately but a whole pool of proteins, also the larger β-barrels – mostly assembled into homo-oligomeric complexes – have their influence. Here, three major factors contribute to the window sizes determined for BSHS225 and BBS275: (i) The β-barrel has a N-terminal and/or C-terminal extension, (ii) one or more long loops break the compact β-barrel domain into two or more parts and (iii) in homo-oligomeric complexes certain parts of the β-barrel domain are involved into protein-protein binding and therefore do not necessarily show an alternating hydrophobicity which results in a smaller scanning window for the BSHS225 compared to the BBS275. Remarkably, according to Wallin and von Heijne [27] most of the in there investigated proteins of eubacterial organisms have a local maximum at six transmembrane helices within a segment of about 225 to 275 residues. The average domain sizes of β-barrel and above mentioned helical membrane proteins lie within the same range. Therefore, the best discrimination between the two structurally different classes might be possible within this domain. This finding further supports our approach to identify β-barrel membrane proteins by searching for the transmembrane domain only.

Subsequently, scores for β-barrel membrane protein prediction were developed using test pools (Fig. 4, Table 2) and three preceding rules. First, a selected protein has to be larger than 80 amino acids, since the smallest monomeric transmembrane β-barrel structure consists of 8 strands [38]. Second, if more than one transmembrane helix is identified by TMHMM, the current protein is not considered as a transmembrane β-barrel protein (Fig. 1) and finally, all scores calculated for a sequence have to be larger than zero, regardless of a performed individual or combined selection. In comparison to the previous procedure [15] we achieved a significant increase of the prediction performance of the *E. coli *proteome (Fig. 5). Certainly, a factor contributing to this achievement was the greater flexibility of the HPS calculation. Wimley [14] originally set the loop length to a minimum of four amino acids. By this slight simplification, as Deng and co-workers [23] also noticed, some hairpins might be missed, because about 28% of the loops are up to three amino acids short [14]. Thus, we kept the window of 25 amino acids for the HPS calculation, but searched for the start of the second β-strand from position 11 to 25, thereby allowing a loop length of 0 to up to 14 amino acids. However, the discriminative power of the linear predictor is limited by the availability of crystal structures of β-barrel membrane proteins. Although about 20 non-redundant structures of this type are currently available in the PDB, they only represent a few families of the diverse group of β-barrel membrane proteins. For example, the important family of β-barrel shaped polypeptide transporters [35] is still missing. A crystal structure of a member of this family would certainly help to improve the predictive power.

In terms of the prediction performance on the sequence pools we have met our goal of reducing the high false negative rate reported by Deng and co-workers [23] for Wimley's [14] original method. Deng and co-workers [23] developed a HMM for discriminating β-barrel membrane proteins. For screening proteomes they raised the threshold score in order to increase the chance of true positive hits. For our procedures we included in the development of the prediction parameters an optimization for a proteome wide scan by taking care of the proposed *in vivo *ratio of OM proteins to NOM proteins. Thus, a direct comparison of the performance on proteomes regarding the test pool derived parameters is not possible. This raises the question, if test pools alone are sufficient to receive an impression of the prediction performance on real proteomes. Regarding the generation of test pools not only a broad and diverse collection of proteins but especially the algorithm to reduce the redundancy of the gathered sequences is of central importance. To keep or not to keep a protein – this is here the question. However, there is possibly still a need for improvement of such redundancy removal algorithms. As a consequence, we suggest testing β-barrel membrane protein prediction procedures also on a real proteome. The very well annotated proteome of the prokaryote *E. coli *[39] is a good candidate for such a model proteome. This additional testing gives the user a better impression of the reliability of the predictions for prokaryotic proteomes and would allow a better comparison of the scores developed.

The combination of different independent procedures for β-barrel membrane protein prediction [18,19] was employed to improve the prediction quality. In here we have analyzed and compared several programs and program combinations. These programs can be classified according to their training sets, to the mathematical procedure taken as basis for the prediction or the size restriction for the sequence analysis window. Therefore, the combination of these programs could be achieved based on the difference of one of the named properties. However, we found that predicting sequences with programs differing in the size restriction for the sequence analysis window revealed the lowest false positive rate based on the *E. coli *proteome. We therefore speculate that the prediction of β-barrel membrane proteins could be further improved employing knowledge based limitations toward the domains, which have to be identified, and global selection approaches in combination.

## Methods

### Test pool generation

In order to evaluate the prediction, the following sequence pools were created with a redundancy of maximal 50%. From the TMPDB [40] databank we retrieved the file TMPDB_alpha_nr_PR.dat [41] which contains a set of α-helical transmembrane proteins. We further collected all OM proteins from the experimentally verified ePSortdb dataset v2.0 [42] and removed proteins that are clearly no integral OM proteins and proteins marked as hypothetical. From the same databank all available proteins of the cytoplasmic membrane and the cytoplasm of Gram-negative bacteria were downloaded.

From PDB [43,44] (version 01/11/2005) we retrieved globular proteins. By SCOP [45] classification we downloaded from PDB proteins with transmembrane helices and all available transmembrane β-barrels.

As mentioned above, we removed all proteins with more than one transmembrane helix predicted by TMHMM and with less than 80 amino acids. Of the initially 1.235 proteins, 782 survived these steps.

All proteins that are not β-barrel membrane proteins and are not from PDB were accumulated in one sequence pool. They are referred to as PSort NOM protein pool. The OM proteins of PSort and PDB were kept each in separate pools.

### Proteome

For testing our in here developed procedure on a real proteome, the genomic derived sequences deposited at [46] (from 01/07/2005) for *E. coli *were used.

### Definition of the scores

The algorithms for EBSS, HPS, BBS and BSN were previously described [14,15]. In brief: The EBSS gives the TM beta-strand probability within a 10 aa sliding window which corresponds to the average length of a TM beta-strand [14]. Approximately 6 aa are required to cross the hydrophobic core of the membrane and further 3 aa for the lipid head group regions of each membrane leaflet [14]. Collecting crystal structures of all β-barrel membrane proteins available in 2002 at a maximum sequence identity of 50% Wimley [14] calculated the statistical occurrence of amino acids belonging to TM β-strands in the above mentioned membrane regions further differentiating between amino acids exposed to the membrane or oriented towards the interior of the pore. Taking into account the typical β-barrel architecture, the HPS is derived by applying a sliding window calculation to the EBSS values. The HPS is calculated by summing up the greatest EBSS of the first 10 and the following 15 residues. The BBS is calculated by adding all HPS values above 6 normalized to the amino acid number of the sequence.

### Calculation of the scores

The BSHS value is derived by calculating the individual score for the β-strand starting at amino acid z. The β-strand position was assigned by the in here redesigned BSN algorithm.

Xstrand(aa=z)=∑i=04(<H>aa(z+(i*2+1))−<H>aa(z+(i*2)))/10     (E1)
 MathType@MTEF@5@5@+=feaafiart1ev1aaatCvAUfKttLearuWrP9MDH5MBPbIqV92AaeXatLxBI9gBaebbnrfifHhDYfgasaacH8akY=wiFfYdH8Gipec8Eeeu0xXdbba9frFj0=OqFfea0dXdd9vqai=hGuQ8kuc9pgc9s8qqaq=dirpe0xb9q8qiLsFr0=vr0=vr0dc8meaabaqaciGacaGaaeqabaqabeGadaaakeaacqqGybawdaWgaaWcbaGaee4CamNaeeiDaqNaeeOCaiNaeeyyaeMaeeOBa4MaeeizaqMaeiikaGIaeeyyaeMaeeyyaeMaeyypa0JaeeOEaONaeiykaKcabeaakiabg2da9maaqahabaGaeiikaGIaeeipaWJaeeisaGKaeeOpa4ZaaSbaaSqaaiabbggaHjabbggaHjabcIcaOiabbQha6jabgUcaRiabcIcaOiabbMgaPjabcQcaQiabikdaYiabgUcaRiabigdaXiabcMcaPiabcMcaPaqabaaabaGaeeyAaKMaeyypa0JaeGimaadabaGaeGinaqdaniabggHiLdGccqGHsislcqqG8aapcqqGibascqqG+aGpdaWgaaWcbaGaeeyyaeMaeeyyaeMaeiikaGIaeeOEaONaey4kaSIaeiikaGIaeeyAaKMaeiOkaOIaeGOmaiJaeiykaKIaeiykaKcabeaakiabcMcaPiabc+caViabigdaXiabicdaWiaaxMaacaWLjaWaaeWaaeaacqqGfbqrcqaIXaqmaiaawIcacaGLPaaaaaa@6E37@

using the water/octanol transfer free energy scale [47], multiplying the EBSS value

Y_strand(aa=z) _= |X_strand (aa=z)_| * EBSS_(aa=z) _    (E2)

adding all values in a defined amino acid window (w) and normalize to that window

BSHS(w)=∑aa=1w−9Ystrand(aa)/w     (E3)
 MathType@MTEF@5@5@+=feaafiart1ev1aaatCvAUfKttLearuWrP9MDH5MBPbIqV92AaeXatLxBI9gBaebbnrfifHhDYfgasaacH8akY=wiFfYdH8Gipec8Eeeu0xXdbba9frFj0=OqFfea0dXdd9vqai=hGuQ8kuc9pgc9s8qqaq=dirpe0xb9q8qiLsFr0=vr0=vr0dc8meaabaqaciGacaGaaeqabaqabeGadaaakeaaieaacqWFcbGqcqWFtbWucqWFibascqWFtbWudaWgaaWcbaGaeiikaGIaee4DaCNaeiykaKcabeaakiabg2da9maaqahabaGaeeywaK1aaSbaaSqaaiabbohaZjabbsha0jabbkhaYjabbggaHjabb6gaUjabbsgaKjabcIcaOiabbggaHjabbggaHjabcMcaPaqabaaabaGaeeyyaeMaeeyyaeMaeyypa0JaeGymaedabaGaee4DaCNaeyOeI0IaeGyoaKdaniabggHiLdGccqGGVaWlcqqG3bWDcaWLjaGaaCzcamaabmaabaGaeeyrauKaeG4mamdacaGLOaGaayzkaaaaaa@54D1@

All scores were described earlier [15]. For transmembrane helix prediction TMHMM v. 2.0 was used [26,48].

## Abbreviations

aa, amino acids; BBS, β-barrel score; BSN, β-strand number; EBSS, exact β-strand score; HPS, hairpin score; BSHS, β-strand based hydrophobicity score; HMM, Hidden Markov Model; MCM, Markov Chain Model; OM, outer membrane; NOM, non-outer membrane
